# Preconception health and care (PHC)—a strategy for improved maternal and child health

**DOI:** 10.1080/03009734.2016.1191564

**Published:** 2016-06-17

**Authors:** Anna Berglund, Gunilla Lindmark

**Affiliations:** a National Centre for Knowledge on Men’s Violence against Women, Uppsala University, Uppsala, Sweden;; b International Maternal and Child Health, Uppsala University, Uppsala, Sweden

**Keywords:** Intimate partner violence, maternal and child health, preconception care, provision of care

## Abstract

Maternal health status before pregnancy is a decisive factor for pregnancy outcomes and for risk for maternal and infant complications. Still, maternity care does not start until the pregnancy is established and in most low-income settings not until more than half of the pregnancy has passed, which often is too late to impact outcomes. In Western societies preconception care (PCC) is widely recognized as a way to optimize women’s health through biomedical and behavioural changes prior to conception with the aim of improving pregnancy outcomes. But the content of PCC is inconsistent and limited to single interventions or preconception counselling to women with chronic illnesses. It has been suggested that PCC should be extended to preconception health and care (PHC), including interventions prior to pregnancy in order to optimize women’s health in general, and thereby subsequent pregnancy outcomes, the well-being of the family, and the health of the future child. With this definition, almost every activity that can improve the health of girls and women can be included in the concept. In the World Health Report of 2005 a longitudinal approach to women’s wellness and reproductive health was highlighted, and the World Health Organization has proposed a more comprehensive maternal and child health care, also including psychosocial issues and intimate partner violence. The present article gives an overview of the recent literature and discusses contents and delivery of PCC/PHC in Western as well as low-income countries. The article puts special emphasis on why violence against women is an issue for PHC.

## Introduction

At the beginning of the new millennium, world leaders gathered at the United Nations to shape a broad vision to fight poverty in its many dimensions, resulting in the eight Millennium Development Goals (MDGs). The stipulated time for realizing the goals expired at the end of 2015. Preliminary results show that the framework has in many ways been successful in focusing on crucial issues for a positive development of global health and welfare. Child mortality for under-five-year-olds was reduced from 90 to 43 deaths for every 100,000 children (MDG 4). Maternal mortality rate has declined by 45% globally. More women have got access to reproductive care, and the use of contraception has increased among married or co-habiting women (MDG 5). However, reproductive health risks are still unacceptably high in many countries, and reproductive health and rights remain one of the most important issues for future development (1).

Maternal health status before pregnancy is a decisive factor for pregnancy outcomes and for the relative risk for maternal as well as infant complications. Still, maternal health care does not start until the pregnancy is well established and in most low-income settings not until more than half of the pregnancy has passed. The term preconception care (PCC) is widely used for activities intended to address and prevent specific problems related to pregnancy. Preconception health and care (PHC), on the other hand, can be defined as the provision of biomedical and behavioural interventions prior to pregnancy in order to optimize women’s wellness and subsequent pregnancy outcomes with the aim to improve not only foetal, infant, and maternal health, but also the health of the whole family and the future well-being of the offspring. Hence PHC includes medical as well as educational and psychosocial issues. With this broad definition, almost every activity that can improve the health of girls and young women can be included in the concept. The present article gives an overview of the recent literature and important issues in the contents and delivery of health care prior to pregnancy in Western as well as low-income countries. The article puts special emphasis on intimate partner violence (IPV) and why violence against women is an issue for PHC.

## The content of preconception health services

In Western societies PCC is widely recognized as a way to optimize women’s health and improving pregnancy outcomes. But although there is a growing body of evidence supporting that very early pregnancy is a critical period for both maternal and foetal health, preconception care is inconsistent, and PCC is mostly limited to single interventions or preconception information and counselling to women with chronic illnesses such as diabetes even in countries with comprehensive health care systems (2). Such programmes are common in many Western countries and have also been assessed as effective. Other initiatives are focused on lifestyle changes such as the cessation of smoking and drinking alcohol, or the supplementation with folic acid.

In order to address the lack of consensus regarding the content and the delivery of PCC in the Netherlands, the health authorities organized an expert meeting. The consensus meeting resulted in the following definition:

A set of interventions and/or programmes that aims to identify and enable informed decision-making to modify biomedical, behavioural, and (psycho-) social risks to parental health and the health of their future child, through counselling, prevention and management, emphasizing those factors that must be acted on before conception and in early pregnancy, to have maximal impact and/or choice (3). This definition of PCC is in accordance with the wider concept of PHC.

Health care in order to improve pregnancy outcomes has rarely been provided in poor countries, where women have many health problems. Even though maternal and child health is traditionally organized together and often provided by the same professional staff, little or no attention is given to the women before and between pregnancies. In the World Health Report, *Make Every Woman and Child Count* (from 2005), the longitudinal approach to women’s wellness and reproductive health was highlighted, and the World Health Organization (WHO) has in many of its programme recommendations proposed a continuum of maternal and child health, and called for a reformulation of interventions from vertical programmes to those offering a wider range of services (4).

In countries where many women have poor health and malnutrition, the activities to be considered for preconception care are numerous. In 2012, WHO organized a meeting to develop a global consensus on preconception care to reduce maternal and childhood mortality and morbidity (5). In a review prepared for the meeting, the list of activities included:

- Tobacco use prevention and cessation programmes
- Nutrition programmes
- Vaccine programmes
- Fertility and infertility programmes
- Female genital mutilation programmes
- HIV testing and counselling programmes
- Mental health programmes
- Substance use programmes
- Intimate partner and sexual violence programmes
- Premarital counselling programmes
- Genetic counselling programmes
- Maternal and child health programmes
- Adolescent-friendly services
- Occupational health programmes

Most of these programmes exist as projects or vertical interventions also in many low-income countries, but not with a perspective of preconception care. Probably, the easiest interventions to introduce are vaccinations such as against rubella, hepatitis, and tetanus, which can be included in general vaccination programmes. Also HIV programmes are often given priority in the health care system, although the barriers for women to attend them are high. Preconception interventions that involve community participation are much more complicated and difficult to implement.

It is often difficult to assess the effects of preventive activities, especially of a general nature. There are many descriptive reports about various projects for preconception information and mostly specific single interventions, but what is the evidence for preconception care being effective in improving pregnancy outcomes? In a recent review only eight randomized studies of moderate to poor quality were found in a literature search, and half of them were single-risk interventions (6). The outcomes assessed were limited to maternal knowledge, self-efficacy and health locus of control, and risk behaviour, with some evidence of improvement. Thus the evidence for improved pregnancy outcomes is indirect at best. The best evidence for important benefits of preconception care is for diabetic women, for whom good metabolic control before pregnancy will decrease the risk of malformations (7).

A comprehensive PHC approach would address both women and men. It should include promotion of a healthy lifestyle before as well as in between pregnancies, such as advice on nutrition to avoid under-nourishment or hazardous overweight and obesity. Appropriate spacing of pregnancies is important to restore a good health status after birth and adequate nutrition of the newborn. PHC also includes education about use of tobacco, alcohol, and drugs and the adverse effects on health and reproduction; safe sex information and education; and family planning including meaningful education about contraception, and discussions about when to start a family. PHC should recognize the importance of avoiding adolescent pregnancies. It is estimated that 60 million adolescents give birth each year worldwide, even though globally pregnancy in adolescence has mortality rates at least twice as high as pregnancy in women aged 20–29 years (8). The issue of gender-based violence and abuse is intertwined in all mentioned aspects and should be appropriately addressed early as well as later (3,5,7).

## Why violence against women is an issue for PHC

According to the Millennium Development Goals Report, progress has been made towards women’s and girls’ equality in education, employment, and political representation over the last two decades. But to achieve universal realization of gender equality and empowerment of women, one of the key areas to address is gender-based discrimination and violence against women and girls (1).

### Prevalence estimates for violence against women

In 2013, WHO published a systematic review of prevalence estimates of intimate partner violence (IPV) against women, and sexual violence victimization from non-partners. In total 77 studies from 56 countries representing nearly 350,000 women, 15 years or older, were included in the review (9). The dominating databases were the WHO Multi-country Study on Women’s Health and Domestic Violence against Women, including women in low- and middle-income countries, the International Violence against Women Survey initiated by the UN in the 1990s, and GENACIS, Gender, Alcohol and Culture, with data from the Americas, Australia and New Zeeland, India, Central Asia, and Europe. The results of the review show that 35% of women worldwide had been subjected to physical or sexual violence by an intimate partner, or sexual violence from a non-partner, or both, during their lifetime. Among women who have at some stage had a sexual partner (ever-partnered) 30% had experienced physical and or sexual violence in their relationships. The prevalence was highest in the WHO African, Eastern Mediterranean, and South-East Asia regions, where approximately 37% reported IPV. In the WHO region of the Americas the next highest prevalence was reported, with approximately 30% of women reporting lifetime exposure. Prevalence was lower in the high-income part of the region (23%) and in the European and the Western Pacific regions, where 25% of ever-partnered women reported lifetime IPV experience. Globally, 7% of women reported sexual abuse by someone that was not their partner. In high-income countries the prevalence was higher, close to 13%. Since sexual activity outside marriage in many cultures is taboo, the authors strongly believe that prevalence might be underestimated. Also, there are very few studies on systematic sexual violence in wartime (9).

In a prevalence study among the 28 member states in the European Union 22% of European women had been subjected to physical and/or sexual violence by a partner after the age of 15. Eleven per cent had experienced sexual violence, and 5% had been raped after the age of 15. In addition, 6% had experienced attempted rape (10). Comparing prevalence estimates among countries might be hazardous because of differences in legislation and because the definition of violence varies. However, in Sweden a national survey reported that 20% of women had experienced rape and/or attempted rape during their lifetime. In total, 14% of the women in the study had been physically abused, 7% sexually abused, and 20% emotionally abused by an intimate partner in a lifetime perspective (11).

### Violence victimization and ill health

Several prevalence studies have also considered the possible consequences for health of violence victimization. A number of important health problems have been identified among victims of IPV. The WHO systematic review of reproductive health found that victimized women were 16% more likely to have a low-birth-weight baby, more than twice as likely to have an abortion, and, in some regions, 1.5 times more likely to acquire HIV. Depression was twice as common among victims, as compared to women who had not experienced partner violence (9). Recently published Swedish research shows that violence victimization is associated with mental as well as physical ill health. The association may be even stronger when the perpetrator is a partner and when the victim has been subjected to more than one form of violence, which usually characterizes violence against women in an intimate relationship (11,12).

### Reproductive coercion

Another aspect of IPV is the possible impact on women’s control of their reproduction. In a systematic review concerning interactions between IPV and use of contraception, the authors found several IPV-related factors that influence women’s abilities to protect themselves against unplanned pregnancies. Violence victimization may eliminate the use of contraceptives, earlier experiences of violence associated with a request for contraceptives may discourage any new attempts or simply the fear of violence could hinder women from requesting the use of contraceptives. Relationship imbalances might also impact on the victim’s self-efficacy, resulting in the belief that she is unable to make any autonomous decisions. The reviewed studies reported that a considerable number of women experience reproductive coercion, either in the form of birth control sabotage or pregnancy coercion. Loss of reproductive control should also take forced abortions into consideration, but here research is very limited. The authors suggest that health personnel, especially within family planning, should include questions about IPV and reproductive coercion in their consultations (13).

### Unintended pregnancy and induced abortion in relation to IPV

Unintended pregnancy was recently addressed in a European multi-country cross-sectional study among women in antenatal care. Approximately one-fifth of all women reported their current pregnancy to be unintended. Among women reporting any lifetime abuse, the prevalence of an unintended pregnancy was 25%, and among women reporting recent abuse 39%. Women with a history of any lifetime abuse had significantly higher odds of unintended pregnancy, also after adjusting for confounding factors, with an odds ratio of 1.41 (95% CI 1.23–1.60) for any lifetime abuse and 2.03 (95% CI 1.54–2.68) for recent abuse (14). The prevalence of IPV among women seeking termination of pregnancy was compared to that of women seeking contraceptive counselling in a recent Swedish study. In total, 29% of women wanting termination of pregnancy and 22% in the family planning group reported IPV. Women seeking abortions were significantly more likely to report IPV (adjusted odds ratio 1.6, 95% CI 1.2–2.1), and among women with repeated terminations the prevalence of IPV was 51% (8,15).

### Identifying violence victimization

Health care has a major role in identifying women subjected to violence. Most women find it acceptable or appropriate to be asked (10,16,17). That trustworthy professionals raised the issue of abuse is considered important by victimized women for their future decisions on taking action to improve their situation (18,19). The questions about violence must, however, be put in an empathic way and in private.

In Western societies we often believe that the only way to avoid recurrent partner violence is to end the relationship, which tends to be complicated and sometimes dangerous. In many places in the world this is even more difficult for cultural reasons. Many researchers have suggested that promoting issues of gender equality could be a way of preventing or reducing IPV. There are encouraging examples within programmes for fighting HIV-AIDS (20,21).

## Timing of PHC

PHC directed towards young people should focus on lifestyle and safe sex issues such as contraception, prevention of sexually transmitted infections (STIs), and gender issues including gender-based violence and human rights. That combining education with provision of contraceptives could be effective in reducing unintended pregnancies among adolescents is supported by a Cochrane review (22).

Raising awareness of the hazards of unprotected sex increases the motivation for preventive measures. A model for education about human papilloma virus (HPV) and its association with cervical cancer has been tried in a randomized controlled trial in Swedish school health care. The intervention was based on the Health Belief Model with 30-minute structured information face-to-face about the risk for disease and means of prevention. At follow-up three months later, awareness of the risks had increased significantly in the intervention group. The intervention also had a favourable effect on the views on prevention of HPV infection, and significantly more young women in the intervention group chose to get vaccinated against HPV (23).

Reproductive Life Plan (RPL) is a tool for information and education about reproductive issues. It has been developed to help women to set realistic goals for their reproduction. RPL provides structured ways of discussing matters such as the impact of age on fertility and how to preserve fertility until pregnancy is wanted (24). RPL has recently been introduced and tried in a Swedish family planning setting (25). The midwives found RPL easy to use and very helpful in the dialogue with their patients (26).

Internationally, some examples of premarital counselling have been initiated and sanctioned by national authorities. In China, premarital counselling was mandatory until 2003 and focused especially on genetic counselling and family planning to strengthen the one-child strategy (27). In Iran, premarital information to couples about family planning was introduced to limit population growth (28). In other Middle Eastern countries premarital counselling has mainly focused on avoiding grave forms of thalassemia (29).

## Providers and organization of PHC

In order to create the best possible health status for all women of reproductive age, health care providers must be aware of the significance of the condition they are treating in case the woman becomes pregnant. Chronic diseases often require medication that may have to be changed before or during pregnancy, and the woman must be well informed of this.

Fragmentation of health care is a problem for many patient groups, but especially when interventions that need the participation of several professionals or a change in life perspective. In many countries the main part of reproductive health care is performed by physicians without contact with the primary level of care. Different aspects of reproductive health are also given as vertical programmes by different caregivers. For instance, family planning services are often separated from STI services. The goal of contraception counselling should, however, not exclusively be to prevent unwanted pregnancy, but also to guard fertility until pregnancy is desired. Therefore, there are many advantages with integrating the two. Information and education about safe sex can naturally be included in testing for STI, as well as in counselling about contraception. Abortion counselling and services could also be integrated into the family planning services (30,31). Planning for future pregnancies with appropriate spacing would improve if the staff was also skilled in contraception. Components of health care during pregnancy such as education about a healthy lifestyle, basic testing for anaemia, and immunization could easily be integrated into comprehensive preconception care and counselling.

In most settings worldwide, facilities already exist for antenatal care and well-baby care and could be extended. It would, however, require some reorientation of staff and that primary health care would assume a central position within the system and the community (31). Nurse-midwives have a suitable education for taking on all the suggested components in preconception care and counselling. The use of tools such as a reproductive life plan or other educational tools adapted to the health literacy of the target group can be helpful in initiating discussions about sexuality and reproduction (5,23–25).

One of the many concerns when considering strategies for the implementation of PHC is the resistance from religious, cultural, traditional, and conservative groups in many societies, where even reproductive health care or health information to young and unmarried persons is seen as threatening moral and family values. This applies especially to information about family planning but can also include issues related to pregnancy in general. There are examples that activities have been renamed in respect for religion and traditions, for instance using the term menstrual regulation instead of termination of pregnancy.

### The Swedish example

Sweden has had a favourable development of antenatal care and reproductive health care for women, resulting in extremely low maternal and perinatal mortality and easily accessible services for family planning and counselling about STIs.

Ever since midwifery was recognized as a medical profession and mandatory training was established in the seventeenth century, Swedish midwives have been responsible for normal deliveries. In the 1930s when antenatal care was organized it became a task for the midwives outside the delivery wards. Social aspects were considered important in Swedish antenatal care from the start. The midwives were to make home visits to give advice on hygiene, nutrition, and infant care and to check that the family was prepared for the arrival of the new baby. Later psychosocial support for both parents was emphasized, and classes were held to prepare for parenthood. Screening for cervical dysplasia to prevent cervical cancer was implemented in the 1960s, and the testing procedure was added to the responsibility of the midwives, as well as testing for sexually transmitted infections.

When the law regulating abortion was liberalized in 1975, the subsequent demands for easily accessible contraception counselling led to the inclusion of family planning in the training of the midwives. They were allowed to administer intrauterine devices for contraception, IUDs and prescribe oral contraceptives and became skilled and appreciated advisors. Education about health issues, such as the risks of smoking and using alcohol during pregnancy, and advice about suitable nutrition and exercise to avoid overweight and diabetes were eventually included in antenatal care. In addition, information about lifestyle factors became a part of contraceptive counselling. Youth clinics were organized in the 1970s and are run by midwives. To support the midwives in complicated cases concerning contraception counselling, pregnancy surveillance, or STIs a supervising physician was appointed, often a specialist in obstetrics and gynaecology at the regional hospital.

The goal of this comprehensive model is to strengthen normality in reproductive processes and to empower patients to make informed choices about their sexual and reproductive health. Midwives as counsellors have become very much appreciated, and the vast majority of women in Sweden choose to go to the midwife for reproductive services. One of the strengths of the Swedish system is the excellent continuity. Patients often meet the same staff for issues of safe sex and family planning, pregnancy, STI, and prevention of cervical cancer. Another strength with the Swedish system is that the services are easily accessible and free of charge.

## Conclusion

PCC has mainly been thought of as counselling before or in early pregnancy to improve maternal and infant health. It has often been limited to single interventions or preconception information and counselling to women with chronic illnesses such as diabetes, or focused on lifestyle changes such as cessation of smoking and drinking alcohol, or supplementation with folic acid. Recently it has been suggested that the perspective should be widened to the well-being of the whole family and the offspring, using the concept PHC instead.

PHC covers all reproductive life. Early preconception counselling should aim to achieve the best possible sexual and reproductive health for both for women and men. It should start early with education about healthy lifestyle habits, safe sex, and pregnancy planning. In the preconception period, when pregnancy is being planned, general as well as specific information adjusted to the prerequisites of the individual couple is needed. During pregnancy health education should be given to both parents-to-be, in combination with support to enhance the transition to parenthood. Between pregnancies, PHC aims to restore the nutritional and health status of the mother and the nutrition of the infant and raise the issue of spacing of pregnancies. PHC should be delivered in a comprehensive model and by staff skilled in sexual and reproductive health. PHC should also be free of charge. It is important that gender equality is addressed in the dialogue with both women and men, and that intimate partner violence is recognized as a possible factor in addition to ill health and non-compliance with health advice. To achieve good continuity, we suggest an organization similar to the Swedish model with nurse-midwives as the primary deliverers of care.
